# The influence of race in older adults with infective endocarditis

**DOI:** 10.1186/s12879-020-4881-7

**Published:** 2020-02-17

**Authors:** Ché Matthew Harris, Waseem Khaliq, Aiham Albaeni, Keith C. Norris

**Affiliations:** 1 Department of General Internal Medicine, Johns Hopkins School of Medicine, Division of Hospital Medicine Johns Hopkins Bayview Medical Center, 5200 Eastern Avenue, Baltimore, MD 21224 USA; 2 Department of Medicine, Division of Cardiology, University of Texas Medical Branch, 301 University Boulevard, Galveston, TX 77555-0570 USA; 3 Department of Internal Medicine, Division of Nephrology, David Geffen School of Medicine, University of California Los Angeles, California, Los Angeles USA

**Keywords:** Racial disparities, Endocarditis, Mortality, Large database, Hospitalizations

## Abstract

**Background:**

Age is a risk factor for infective endocarditis, and almost half of diagnosed patients are age ≥ 60 years. Large national studies have not evaluated inpatient mortality and surgical valvular interventions between older White and Black patients hospitalized with infective endocarditis.

**Methods:**

We used the Nationwide Inpatient Sample database to identify older adults ≥60 years in North America with a principle diagnosis of infective endocarditis. Multivariate logistic regression was used to compare in-hospital mortality and valvular repairs/replacement between older Black and White patients.

**Results:**

Of 10,390 adults, age ≥ 60 years hospitalized for infective endocarditis during 2013 and 2014, 7356 were White and 1089 Black. Blacks were younger (mean age: 70.5 ± 0.5 vs. 73.5 ± 0.2 years, *p* < 0.01), lived in more zip codes with a median annual income <$39,000/yr. (40.4% vs 18.8%, *p* < 0.01), and had higher co-morbidity burden (Charlson comorbidity score ≥ 3: 54.6% vs 40.7%, *p* < 0.01). After multivariate adjustment, Blacks had higher odds for in-hospital mortality (Odds Ratio (OR) = 2.0, [Confidence Interval (CI) 1.1–3.8]; *p* = 0.020), and lower odds for mitral valve repairs/replacements (OR = 0.53, CI: 0.29–0.99, *p* = 0.049).

**Conclusions:**

Blacks age ≥ 60 years hospitalized in North America with infective endocarditis are less likely to undergo mitral valvular repairs/replacement and had higher in-hospital mortality compared to White patients.

## Background

Infective endocarditis (IE) is a debilitating, life-threatening infection of heart valves that affects up to 20,000 individuals each year in the United States [1, 2]. With an inpatient mortality rate ranging from 20 to 64%, prompt recognition and timely intervention to treat IE is critical to patient survival [3, 4]. Older age (≥60 years) confers an increased risk for IE [5] and almost half of affected individuals are in this age category [6]. Given the growing life expectancy in the United States, an increase in incidence of IE can be expected among the older population. *Staphylococcus aureus* is the leading bacterial organism found in patients with IE, and higher risks for invasive Methicillin Resistant *Staphylococcus aureus* (MRSA) infections have been reported in Black patients as compared to their White counterparts [7].

Moreover, statewide database analysis has described racial disparities in infectious disease complications that continue to plague Black patients [8], though the impact of IE, specifically is not entirely clear. Additionally, valvular repairs/replacements are often indicated for patients who fail to respond appropriately to antimicrobials or develop significant complications from IE. However, prior studies have shown that Black patients receive less aortic and mitral valve repairs compared to White patients when such procedures are indicated [9, 10]. Thus, determining if differences exist for in-hospital mortality and surgical interventions in older patients with IE by race is crucial to understanding potential aspects of care that need improvement. As such, we hypothesized that Black patients age ≥ 60 years with IE would have higher in-hospital mortality and lower rates of valvular interventions. We utilized the National Inpatient Sample (NIS) database to help determine if differences in clinical outcomes existed in older patients with IE based on race.

## Methods

### Setting

This study used pooled data of years 2013 and 2014 collected from NIS. The NIS comes from the Agency for Healthcare Research and Quality as part of the Healthcare Cost and Utilization Project [11], and it is one of the biggest all-payer inpatient care databases in the United States. A 20 % probability sample of all discharges from contributing hospitals is gleaned and data on demographics, medical conditions, length of stay, and hospital charges are incorporated. Discharges are weighted, thus making the database nationally representative. NIS complies with the Health Insurance Portability and Accountability Act, using de-identified data of hospitals, patients and providers. From 2013 to 2014, NIS included over 7 million discharges yearly from 4363 to 4411 hospitals in 44 states across the United States [11].

### Study population

**Fig. 1:**
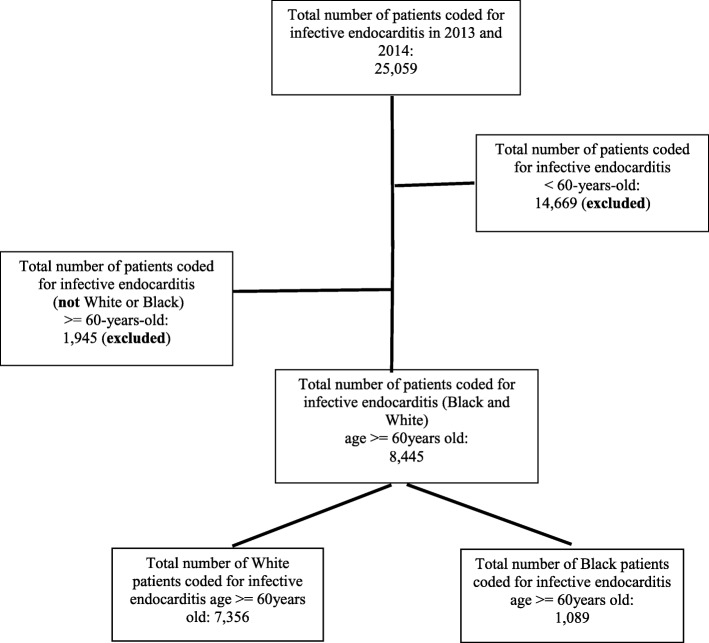
Patient identification flow diagram 2013–2014

Patients were included in the study if they were age ≥ 60 years, had a principal diagnosis of infective endocarditis, and a self-reported racial identifier of either White or Black. Patients were excluded if they were younger than 60 years of age and non-White or non-Black (Fig. 1). The following ICD-9-CM codes were used to identify eligible admissions:

1. Infective endocarditis: 421.0 (acute and subacute bacterial endocarditis), 421.1 (acute and subacute bacterial in diseases classified elsewhere), 421.9 (acute endocarditis, unspecified), 424.9 (endocarditis, valve unspecified), 093.2 (syphilitic endocarditis), 0.9884 (gonococcal endocarditis), 0.74.22 (coxsackie endocarditis), 0.3642 (meningococcal endocarditis), 391.1 (acute rheumatic endocarditis), 112.81 (candida endocarditis), 115.04 (*Histoplasma capsulatum* endocarditis), 115.14 (histoplasma duboisii endocarditis), 115.94 (histoplasma unspecified endocarditis).

### Study outcomes

In-hospital mortality was the primary outcome. Secondary outcomes included combined aortic valve repairs or replacements (ICD-9 35.11, 35.21, 35.22), combined mitral valve repairs (ICD-9 35.12, 35.23, 35.24), or replacements, and combined tricuspid valve repairs or replacements repairs (ICD-9 35.14, 35.27, 35.28).

### Patient and hospital characteristics

The main independent variable was race (White or Black status). Patient and hospital level characteristics were collected and adjusted for in the analyses as potential confounders. These included: 1) patient level variables: age (in years), gender, median household income in the patient’s zip code, insurance and co-morbidities [11]; 2) hospital level variables: hospital bed size, teaching status, urban location, and region. We used the Charlson comorbidity index variable that is able to be downloaded for Stata© [12]. The Charlson comorbidity index variable represents the cumulative rise in likelihood for one-year mortality as a result of the severity of comorbidities, and we used this variable in the model as described for administrative databases for further adjustment [13]. The Johns Hopkins University School of Medicine’s Institutional Review Board determined the study was exempt from approval because it involved retrospective analyses of publicly available de-identified data.

### Statistical analyses

Patient demographics, co-morbidities, and hospital characteristics were compared for the years 2013 and 2014 using Pearson’s χ^2^ test for categorical variables and linear regression (1-way ANOVA) for continuous variables. Means and proportions of outcomes of interest were similarly compared. Univariate analysis focused on in-hospsital mortality and repairs/replacements of aortic and mitral valves. All categorical data were presented as proportions/percentages. Logistic regression was used to calculate unadjusted and adjusted odds ratios comparing inpatient mortality and valve replacement between Black and White patients with IE. Adjusted/multivariate regression models included all variables that were found to be associated with the outcome (such as age, gender, acute renal failure, acute heart failure, drug use, human immunodeficiency virus, sepsis, septic shock, heart block, cardiogenic shock and candidemia) on univariate analysis with a *p*-value cutoff of 0.2. We also included patient and hospital variables that were deemed important determinants of outcomes based on prior studies (including hospital volume, hospital geographic region and academic status).

Conditions such as strokes, acute renal failure, diabetes, heart failure and myocardial infarction were accounted for in the Charlson comorbidity variable. In addition, we included clinical other conditions that could impact the outcome measures in patients with IE (see Supplemental Table S1 for ICD-9-CM codes). The OR/β coefficients and 95% confidence interval were used to report the results of regression models. Following adjustment for age, sensitivity analyses between Black and White adults age 18 years and older, and the results did not differ from primary outcome measures (Supplemental Tables S2 and S3).

We used Stata 15.0 (StataCorp, College Station, TX) to perform all statistical analyses. Analyses accounted for survey design complexity (stratification, clustering, and weighting to produce nationally representative results) based on Healthcare Cost and Utilization Project NIS’ analytic guidelines for incorporating sampling weights, primary sampling units, and strata [11]. The *p* values were 2 sided and type I error set at 0.05.

## Results

In 2013 and 2014 there were 8445 hospitalizations for White and Black adults ≥60 years old with infective endocarditis. There were 7356 White patients and 1089 Black patients (Fig. 1). Black patients were younger compared to White patients (mean age: 70.5 ± 0.5 vs. 73.5 ± 0.2 years, *p* < 0.01), had significantly higher prevalence of living in a zip code with a median income level < $39,000/yr. (40.4% vs 18.8%, *p* < 0.01), higher Charlson comorbidity score ≥ 3: 54.6% vs 40.7%, *p* < 0.01), and higher prevalence of cardiogenic shock (*p* = 0.03), complicated diabetes mellitus (*p* < 0.01), drug use (*p* < 0.01), and history of valvular heart disease (*p* = 0.04) (Table 1). There was a greater prevalence of hospitalization for endocarditis in Southern regions of the United States compared to other regions, and this was relatively higher for Black patients compared to their White peers (42.6% vs 32.3%, *p* = 0.01).

**Table 1:** Demographics and hospital outcomes comparing White and Black patients age ≥ 60 years and older hospitalized with infective endocarditis, National Inpatient Sample (2013–2014)

Patient characteristics and outcomes	White patients *N* = 7356	Black patients *N* = 1089	*p*-value
Characteristics			
Age (years) (mean ± SE)	73.5 ± 0.2	70.5 ± 0.5	< 0.01
Female, n (%)	2692 (36.6)	454 (41.7)	0.15
Charlson comorbidity score, n (%)			< 0.01
0	1353 (18.4)	84 (7.8)	
1	1544 (21.0)	194 (17.8)	
2	1.449 (19.7)	214 (19.7)	
3 or more	2993 (40.7)	594 (54.6)	
Comorbidities, (n%)			
Acute heart failure	1,1,18 (15.2)	184 (16.9)	0.51
Acute renal failure	1691 (23.0)	284 (26.1)	0.33
Candidemia	22 (0.3)	14 (1.3)	0.19
Cardiogenic shock	176 (2.4)	64 (5.9)	0.03
Complicated diabetes mellitus	536 (7.3)	154 (14.2)	< 0.01
Drug use	117 (1.6)	74 (6.8)	< 0.01
History of heart block	397 (5.4)	69 (6.4)	0.59
Human Immunodeficiency Virus	14 (0.2)	14 (1.3)	0.14
History of cerebral vascular accident	1213 (16.5)	199 (18.3)	0.51
Sepsis	926 (12.6)	193 (17.8)	0.47
Septic emboli	684 (9.3)	114 (10.5)	0.59
History of valvular disease	132 (1.8)	24 (2.2)	0.04
Median income in patient’s zip code, n (%)			< 0.01
$1–$38,999	1382 (18.8)	440 (40.4)	
$39,000–$47,999	1780 (24.2)	279 (25.7)	
$48,000–$62,999	1949 (26.5)	228 (20.9)	
$63,000 or more	2228 (30.3)	146 (13.4)	
Insurance type, n (%)			< 0.01
Medicare	5678 (77.2)	815 (74.9)	
Medicaid	213 (2.9)	81 (7.5)	
Private	1360 (18.5)	180 (16.5)	
Uninsured	95 (0.1.3)	10 (0.94)	
Hospital bed size, n (%)			0.09
Small	1059 (14.4)	100 (9.2)	
Medium	1964 (26.7)	278 (25.6)	
Large	4325 (58.8)	709 (65.1)	
Hospital region, n (%)			0.02
Northeast	2118 (28.8)	249 (22.9)	
Midwest	1493 (20.3)	230 (21.1)	
South	2375 (32.3)	464 (42.6)	
West	1353 (18.4)	264 (15.6)	
Teaching hospital, n(%)			< 0.01
Non-teaching	926 (12.6)	61 (5.6)	
Teaching	6429 (87.4)	1028 (94.4)	
Outcomes			
In-hospital mortality, n (%)	448 (6.1)	104 (9.6)	0.09
AVR, n (%)	750 (10.2)	119 (11.0)	0.7
MVR, n (%)	698 (9.5)	95 (8.7)	0.66
TVR, n (%)	44 (0.6)	10 (0.9)	0.6

*n* Number, *AVR* Aortic valve repairs/replacements, *MVR* Mitral valve repairs/replacements, *TVR* Tricuspid valve repairs/replacement

### In-hospital mortality and Valvular repairs/replacements

Unadjusted Analyses: Crude analysis found that the proportion of in-hospital mortality cases for Blacks was higher compared to Whites (9.6% vs 6.1%, *p* = 0.09), but did not reach statistical significance. The proportion of valve repairs/replacements for Black and White patients were as follows: aortic (11.0% vs 10.2%, *p* = 0.7), mitral (8.7% vs 9.5%, *p* = 0.66) and tricuspid (0.9% vs 0.5%, *p* = 0.6). The unadjusted analysis showed no significant difference in White compared to Black populations in-patient mortality (OR = 1.6 [CI = 0.98–2.6]; *p* = 0.057) or repairs/replacements of aortic valves (OR = 1.0 [CI = 0.69–1.7]; *p* = 0.71) mitral valves (OR = 0.9 [CI = 0.56–1.5]; *p* = 0.67) and tricuspid valves (OR = 0.7 [CI = 0.4–7.4]; *p* = 0.36).

Adjusted Analyses: After multivariate adjustment for demographic, clinical and hospital level variables, Black patients had significantly higher odds for in-hospital mortality (OR = 2.0, [(CI) 1.1–3.8]; *p* = 0.022), and lower odds for mitral valve repairs/replacements (OR = 0.53, [(CI) 0.29–0.049]; *p* = 0.020). The odds for all other repairs/replacements of aortic and tricuspid valves did not differ by race (Table 2). When analyzing in-hospital mortality outcome while adjusting for all valvular repairs/replacements, Black patients continued to have a higher odds of death (OR = 2.0, [(CI) 1.1–3.8]; *p* = 0.020).

**Table 2:** Odds ratios for hospital outcomes in infective endocarditis for black and white patients

Racial status (2013 and 2014)	Multi-variable Un-adjusted Odds Ratio (95% CI)	*p*-value	Multi-variable Adjusted Odds Ratio (95% CI)	*p*-value
Valve replacement treatment				
Mitral valve replacement (MVR)				
White patients (ref)	**1.0**		**1.0**	
Black patients	**0.9 (0.56–1.58)**	**0.67**	**0.51 (0.27–0.95)**	**0.036**
Aortic valve replacement (AVR)				
White Patients (ref)	**1.0**		**1.0**	
Black patients	**1.0 (0.69–1.7)**	**0.71**	**0.82 (0.47–1.5)**	**0.46**
Tricuspid valve replacement (TVR)				
White patients (ref)	**1.0**		**1.0**	
Black patients	**0.7 (0.4–7.4)**	**0.3**	**2.3 (0.29–19.6)**	**0.40**
In-hospital mortality				
White patients (ref)	**1.0**		**1.0**	
Black patients	**1.6 (0.98–2.6)**	**0.057**	**2.0 (1.1–3.8)**	**0.020**

*ref* Reference, *CI* Confidence Interval. The multi-variable analysis adjusted for the following: age, gender, median household income, insurance and Charlson comorbidity index and additional clinical factors that differed between groups with a *p* value of < 0.2 that included cardiogenic shock, cardiac arrest, hospital bed size, teaching status, urban location, and region

## Discussion

Our study is one of the first to evaluate racial differences in in-hospital clinical outcomes between Black and White patients age ≥ 60 years diagnosed with IE in the United States. We found that Black patients had significantly higher in-hospital mortality and lower mitral valvular repairs/interventions compared to White patients. With more Americans making up a growing portion of older adults, and because older age is a risk factor for IE, it is anticipated that this population will contribute to more acute hospitalizations in the years to come. We initially appreciated a difference in mortality between Black and White patients age ≥ 18 years and older, but after adjustment for age, we did not appreciate the mortality difference, which prompted investigation into older adults with IE. Awareness of disparities in this already vulnerable population of older adults is critical to reduce morbidity and mortality from IE. In addition, though improvements in racial disparities in healthcare for older adults has occurred overtime, it has also been observed that there remains room for improvement [14]. This study highlights an underrecognized disparity in clinical outcomes for older Black patients with IE, and may help guide future research to better determine etiologies to target for intervention.

The higher mortality in Black patients over White patients impacted by IE is not unexpected especially given risk factors that Black patients are known to have more cases of drug resistance organisms [7]. Gualandi and colleagues found a longstanding, unwavering disparity of a higher number Blacks with MRSA compared to White patients between the years 2004 to 2015 [7]. Though our study did not investigate which organisms were isolated in IE cases, we suspect that more Black patients might have had MRSA when compared to their White counter parts; hence, rendering management of IE difficult. Future studies may need to target which organisms are more common in Black patients and White patients with IE. Our study found that after adjusting for multiple variables, such as demographics, income, comorbidities and facility level characteristics, Black race was independently associated with higher mortality in older patients. The finding of this health disparity in Black patients is consistent with similar findings for many health conditions in the literature in the United States [15]. However, observational studies cannot determine causality and further investigation is needed to better understand why older Blacks with IE have higher mortality than Whites.

Research conducted by Liu et al. found that Blacks were less likely than Whites to receive care at high-volume hospitals that carry out complex surgical procedures, such as valve replacements [16]. Further, using national Medicare data, Dimick et al. found that Black patients were more likely than Whites to have high risk surgeries at lower quality hospitals in certain geographical regions [17]. Our study could not determine volume or quality of given hospitals, but only appreciated lower rates of mitral valvular interventions for Black patients. Our finding is consistent with research conducted by DiGiorgi, who noted that Black patients have less mitral valve surgeries compared to White patients [10]. Though DiGiorgi didn’t specifically study older adults, Blacks also presented at a younger age for mitral valvular intervention and had more comorbidities than White patients, which was similar to our findings. The reason(s) Blacks have lower rates of mitral valve interventions is not clear, and though they had more comorbidities than Whites in our study, following adjustment for this factor, Blacks continued to have lower mitral valve interventions and higher mortality.

This is critical because research has shown that the mitral valve is the most common valve affected by endocarditis [18]. The lower mitral valve interventions and higher mortality rates in Black patients in our study might suggest that lack of surgical intervention preceded mortality. However, our observational study was not able to determine this. That said, there was no significant difference in aortic valve repairs/replacements in our study, and the aortic valve is the second most common valve affected by IE [18]. In another study using the NIS that compared aortic valve replacements in Black and White patients who presented with aortic stenosis, researchers found that Blacks were less likely to undergo surgery, as suggested by Alqahtani and Patel [9, 19]. One potential reason the authors concluded for this finding was that Black patients are less likely to have severe aortic stenosis in general [19]. Our study looked at all older Black and White patients with IE, and we also evaluated both aortic valve repairs and replacements. More studies need to be carried to better determine frequency of differences of aortic valvular interventions in older adults with IE.

Race appeared to be an independent exposure for increased in-hospital mortality and lower mitral valvular interventions in our study. No studies to date have investigated the impact race has had on clinical outcome for IE, and this research may help to bring attention to an under-addressed area in infectious diseases. Given the acute, severe and rapidly devastating consequences of IE, increased cognizance that older Black patients may need closer monitoring and/or aggressive therapeutic intervention to reduce mortality is essential. Increasing awareness of this disparity may help to improve health outcomes in older Black patients. As noted by Wesson, this needs a team effort that involves health systems and their leaders to improve not only care delivery, but improvement in population health outcomes to build community trust and eliminate disparities [20].

This study using NIS had some limitations that are notable to mention. First, NIS is an administrative database dependent on correct incorporation of ICD-9 and ICD-10 CM codes. Errors can arise if codes were not entered or incorporated inaccurately. Thus, the quality of certain variables such as the Charlson index might not have been completely accurate. However, many published studies use administrative databases and this is an unfortunate limitation of these types of data sources.

Also, NIS lacks laboratory and imaging data, and there is no way to determine which medications were given to patients. Granular details of why surgery may or may not have been deferred are also could not be captured. Additionally, the quality of echocardiogram could not be obtained and we were unable to determine detailed information, such as the size of valvular vegetations, presence of perivalvular abscesses or degree of cardiac damage. These echocardiographic findings help determine need for surgical intervention. Another limitation was that unlike some other large databases, NIS can account for race but not ethnicity. Also, NIS cannot precisely account for patients’ current functional, nutritional or cognitive characteristics, as these were recently shown to be key prognostic parameters of IE in elderly patients [21].

Next, due to under-coding of multi-drug resistance organisms, we were not able to adjust for this variable in multi-variate analysis of valvular repairs/replacements and mortality. Also, this study did not look at specific bacterial types involved in IE. Similarly, because fungemia was limited to candida blood stream infections on review of ICD-9 codes, other common fungal organisms in IE, such as aspergillus [22] could not be used in the model. However, because candida is the leading cause of fungemia in IE [23], we suspected that it was an appropriate variable to include. Additionally, it is possible that there may be unmeasured confounders, which can occur with observational studies. That said, we attempted to follow similar studies using the NIS to account for as many relevant variables as possible. Furthermore, NIS is limited to the United States of America, and the global impact of race on IE could not be determined. Inclusion of only adult patients may possibly represent a selection bias, which may affect the results. However, many studies in NIS only study adult populations and we suspect that a significantly higher number of adults are impacted by endocarditis.

The strengths of the study are that NIS represents up to 44 states, and results reflect what can be expected in the larger population. Additionally, NIS is able to take uncommon conditions, such as IE and pool enough affected individuals for general study analysis.

## Conclusions

In conclusion, disparities exist for inpatient mortality and valvular interventions between older Black and White patients with IE. Awareness of these disparities and future efforts to determine corrective measures is crucial to narrowing gaps in patient mortality and surgical outcomes for this vulnerable population. Future studies need to evaluate individual level predictors that influence mortality and treatment for IE and develop strategies to reduce such disparities.

## Supplementary information

**Additional file 1: Table S1.** Additional ICD-9-CM codes used for relevant clinical conditions that could affect endocarditis and mortality.

**Additional file 2: Table S2.** Demographics and hospital outcomes comparing White and Black patients age ≥ 18 and older hospitalized with infective endocarditis, National Inpatient Sample (2013–2014). **Table S3.** Adults age 18 years and older: Odds ratios for hospital outcomes in infective endocarditis for black and white patients.

## Data Availability

Researchers should readily be able to purchase the same databases we did to conduct research here: https://www.distributor.hcup-us.ahrq.gov/Databases.aspx. The authors did not have special access privileges to the NIS databases. Contact information for further guidance on purchase and download at vog.qrha@rotubirtsiDPUCH.

## Abbreviations

*AVR*: Aortic Valve repairs/replacements
*CI*: Confidence Interval
*ICD-10-CM*: International Classification of Diseases, Tenth Revision, Clinical Modification
*ICD-9-CM*: International Classification of Diseases, Ninth Revision, Clinical Modification
*IE*: Infective Endocarditis
*MRSA*: Methicillin Resistant *Staphylococcus aureus*
MVR: Mitral valve repairs/replacements
*n*: Number
*NIS*: National Inpatient Sample
*OR*: Odds *Ratio*
*ref*: Reference
*TVR*: Tricuspid valve repairs/replacements
*TX*: Texas
