# Correction to: The influence of race in older adults with infective endocarditis

**DOI:** 10.1186/s12879-020-04970-3

**Published:** 2020-03-24

**Authors:** Ché Matthew Harris, Waseem Khaliq, Aiham Albaeni, Keith C. Norris

**Affiliations:** 1 Department of General Internal Medicine, Johns Hopkins School of Medicine, Division of Hospital Medicine Johns Hopkins Bayview Medical Center, 5200 Eastern Avenue, Baltimore, MD 21224 USA; 2 Department of Medicine, Division of Cardiology, University of Texas Medical Branch, 301 University Boulevard, Galveston, TX 77555-0570 USA; 3 Department of Internal Medicine, Division of Nephrology, David Geffen School of Medicine, University of California Los Angeles, Los Angeles, CA USA

**Correction to: BMC Infect Dis**

**https://doi.org/10.1186/s12879-020-4881-7**

After publication of the original article [1], there is a duplicate “35.22” in the section “Study outcomes”: Secondary outcomes included combined aortic valve repairs or replacements (ICD-9 35.11, 35.22, 35.22), […]”. This should be read “(ICD-9 35.11, 35.21, 35.22)”, instead.

The original article has been corrected.
