# Supplementary material for: The influence of race in older adults with infective endocarditis
Source: BMC Infect Dis. 2020 Feb 17;20:146. doi: 10.1186/s12879-020-4881-7 (PMC7027119; doi:10.1186/s12879-020-4881-7)
Supplement: Supplementary file 1 — Additional file 1: Table S1. Additional ICD-9-CM codes used for relevant clinical conditions that could affect endocarditis and mortality. [file 12879_2020_4881_MOESM1_ESM.docx]

**Supplemental Table 1** Additional ICD-9-CM codes used for relevant clinical conditions that could affect endocarditis and mortality

| **Conditions or indications for valve intervention** | **ICD-9 diagnosis or procedure codes** |
| --- | --- |
| Candidemia | 112.5, 112.89 |
| Heart block | 426.0, 426. 6, 426.9, 426. 11, 426.12, 426.13 |
| Septic emboli | 415.12, 449 |
| **In-hospital mortality** |  |
| In-hospital cardiac arrest | 99.60, 99.63 |

Abbreviations: ICD-9-CM: International Classification of Diseases, Ninth Edition Clinical Modification.
