# Supplementary material for: The influence of race in older adults with infective endocarditis
Source: BMC Infect Dis. 2020 Feb 17;20:146. doi: 10.1186/s12879-020-4881-7 (PMC7027119; doi:10.1186/s12879-020-4881-7)
Supplement: Supplementary file 2 — Additional file 2: Table S2. Demographics and hospital outcomes comparing White and Black patients age ≥ 18 and older hospitalized with infective endocarditis, National Inpatient Sample (2013–2014). Table S3. Adults age 18 years and older: Odds ratios for hospital outcomes in infective endocarditis for black and white patients. [file 12879_2020_4881_MOESM2_ESM.docx]

**Supplementary Table 2: Demographics and hospital outcomes comparing White and Black patients** age ≥18 **and older hospitalized with infective endocarditis, National Inpatient Sample (2013-2014)**

| **Patient characteristics and outcomes** | **White patients**  **N = 17,280** | **Black patients**  **N = 2,905** | **p-value** |
| --- | --- | --- | --- |
| **Characteristics** |  |  |  |
| Age (years) (mean $\pm$ SE) | 54.9 $\pm$ 0.4 | 55.3 $\pm$ 0.6 | 0.63 |
| Female, n (%) | 6,583 (38.1) | 1,034 (35.6) | 0.28 |
| Charlson comorbidity score, n (%) |  |  | <0.01 |
| 0 | 5,719 (33.1) | 238 (8.2) |  |
| 1 | 4,008 (23.2) | 505 (17.4) |  |
| 2 | 2,678 (15.5) | 557 (19.2) |  |
| 3 or more | 4,855 (28.1) | 1,365 (47.1) |  |
| Comorbidities, n(%) |  |  |  |
| Acute heart failure | 1,831 (10.6) | 424 (14.6) | <0.01 |
| Acute renal failure | 3,283 (19) | 673 (23.2) | 0.02 |
| Candidemia | 155 (0.9) | 29 (1.0) | 0.9 |
| Cardiogenic shock | 535 (3.1) | 127 (4.4) | 0.13 |
| Complicated diabetes mellitus | 933 (5.4) | 354 (12.2) | <0.01 |
| Drug use | 4,613 (26.7) | 604 (20.8) | 0.02 |
| History of heart block | 898 (5.2) | 214 (7.4) | 0.08 |
| Human Immunodeficiency Virus | 103 (0.6) | 133 (4.6) | <0.01 |
| History of cerebral vascular accident | 2,384 (13.8) | 383 (13.2) | 0.67 |
| Sepsis | 2,367 (13.7) | 377 (13.0) | 0.65 |
| Septic emboli | 3,456 (20.0) | 424(14.6) | <0.01 |
| History of valvular disease | 276 (1.6) | 87 (3.0) | 0.05 |
| Median income in patient’s zip code, n (%) |  |  | <0.01 |
| $1-$38,999 | 4,700 (27.2) | 1,362 (46.9) |  |
| $39,000-$47,999 | 4,354 (25.2) | 656 (22.6) |  |
| $48,000-$62,999 | 3,974 (23.0) | 546 (18.8) |  |
| $63,000 or more | 4,129 (23.9) | 334 (11.5) |  |
| Insurance type, n (%) |  |  | <0.01 |
| Medicare | 7,361 (42.6) | 1,406 (48.4) |  |
| Medicaid | 3,525 (20.4) | 714 (24.6) |  |
| Private | 4,596 (26.6) | 607 (20.9) |  |
| Uninsured | 1,779 (10.3) | 197 (6.8) |  |
| Hospital bed size, n (%) |  |  | 0.08 |
| Small | 2,315 (13.4) | 284 (9.8) |  |
| Medium | 4,354 (25.2) | 737 (25.4) |  |
| Large | 10,592 (61.3) | 1,879 (64.7) |  |
| Hospital region, n (%) |  |  | <0.01 |
| Northeast | 4,199 (24.3) | 572 (19.7) |  |
| Midwest | 3,248 (18.8) | 543 (18.7) |  |
| South | 6,963 (40.3) | 1,472 (50.7) |  |
| West | 2,851 (16.5) | 307 (10.6) |  |
| Teaching hospital, n (%) |  |  | <0.01 |
| Non-teaching | 2,160 (12.5) | 101 (3.5) |  |
| Teaching | 15,120 (87.5) | 2,800 (96.4) |  |
| Outcomes |  |  |  |
| In-hospital mortality, n (%) | 777 (4.5) | 194 (6.7) | 0.01 |
| AVR, n (%) | 2,090 (12.1) | 429 (14.8) | 0.08 |
| MVR, n (%) | 2,004 (11.6) | 429 (14.8) | 0.03 |
| TVR, n (%) | 449 (2.6) | 90 (3.1) | 0.5 |

n = number; AVR = aortic valve repairs/replacements; MVR = mitral valve repairs/replacements;

TVR = tricuspid valve repairs/replacement

**Supplemental Table 3. Adults age 18 years and older:** Odds ratios for hospital outcomes in infective endocarditis for black and white patients

| **Racial status (2013 and 2014)** | **Multi-variable Un-adjusted**  **Odds Ratio (95% CI)** | **p-value** | **Multi-variable Adjusted**  **Odds Ratio (95% CI)** | **p-value** |
| --- | --- | --- | --- | --- |
| **Valve replacement treatment** |  |  |  |  |
| Mitral valve replacement (MVR) |  |  |  |  |
| White patients (ref) | 1.0 |  | 1.0 |  |
| Black patients | 1.3 (1.0-1.67) | 0.03 | 1.1 (0.85-1.5) | 0.35 |
| Aortic valve replacement (AVR) |  |  |  |  |
| White Patients (ref) | 1.0 |  | 1.0 |  |
| Black patients | 1.3 (0.96-1.6) | 0.09 | 0.89 (0.65-1.2) | 0.46 |
| Tricuspid valve replacement (TVR) |  |  |  |  |
| White patients (ref) | 1.0 |  | 1.0 |  |
| Black patients | 1.1 (0.7-1.9) | 0.5 | 2.3 (0.29-19.6) | 0.40 |
| **In-hospital mortality** |  |  |  |  |
| White patients (ref) | 1.0 |  | 1.0 |  |
| Black patients | 1.5 (1.1-2.2) | 0.02 | 1.5 (0.9-2.3) | 0.09 |
